# Supplementary material for: The interactome of intact mitochondria by cross-linking mass spectrometry provides evidence for coexisting respiratory supercomplexes
Source: Mol Cell Proteomics. 2017 Dec 8;17(2):216–32. doi: 10.1074/mcp.RA117.000470 (PMC5795388; doi:10.1074/mcp.RA117.000470)
Supplement: Supplemental Data [file supp_RA117.000470_133922_0_supp_23978_fzffwf.zip › spectra_annotation/mito_DR_spectra_annotation/187-1-4-1-3-1.pdf]

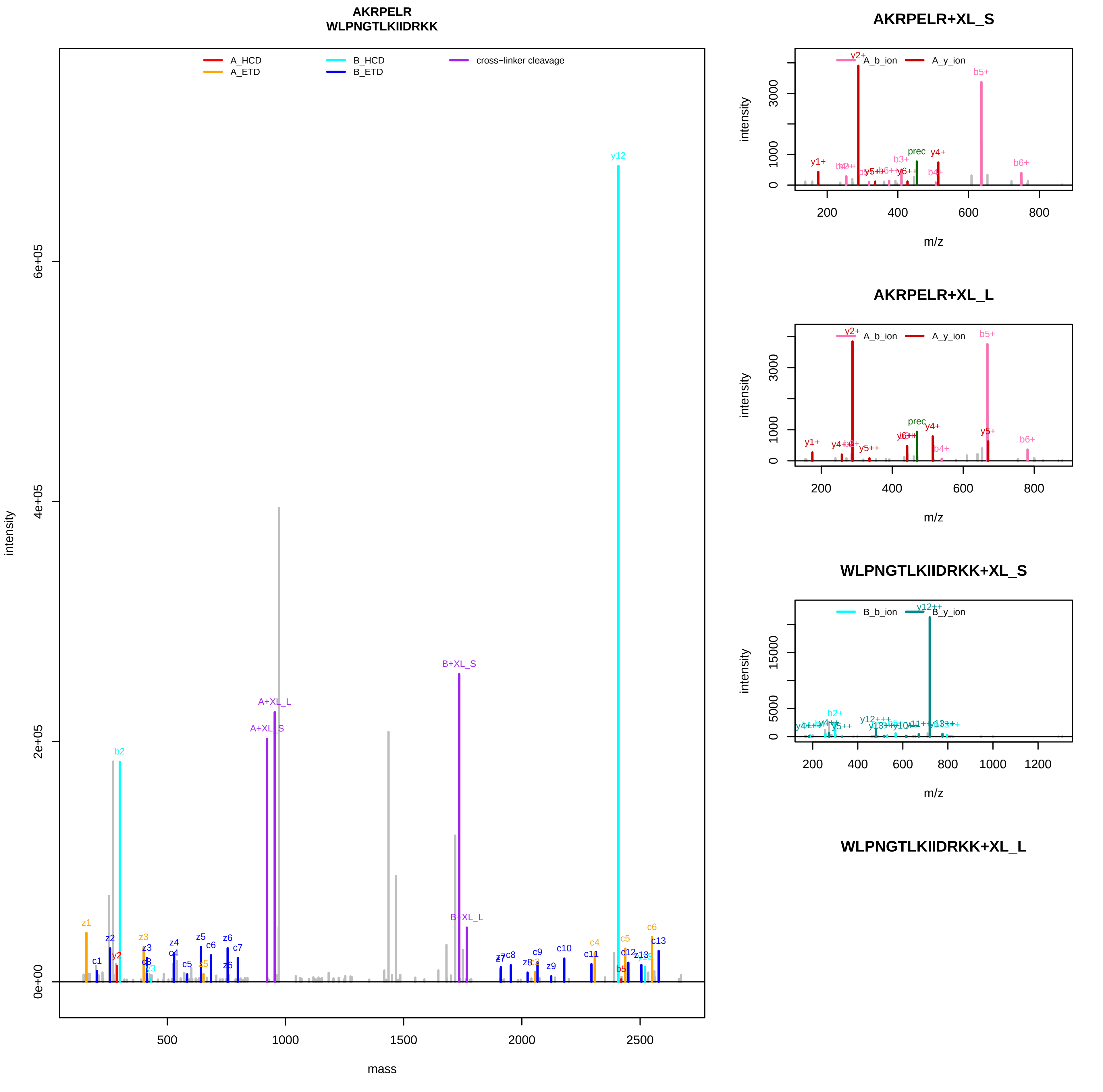
AKRPELR
WLPNGTLKIIDRKK
A_HCD
A_ETD
B_HCD
B_ETD
cross−linker cleavage
intensity
mass
0e+00
2e+05
4e+05
6e+05
500
1000
1500
2000
2500
y12
b2
A+XL_L
A+XL_S
B+XL_S
B+XL_L
z1
c1
z2
y2
z3
z4
c4
z5
c5
c6
z6
c7
z7
c8
z8
c9
z9
c10
c11
c4
c5
c12
z13
b5
c6
c13
AKRPELR+XL_S
AKRPELR+XL_L
WLPNGTLKIIDRKK+XL_S
WLPNGTLKIIDRKK+XL_L
A_b_ion
A_y_ion
B_b_ion
B_y_ion
m/z
prec
y1+
y2+
y4+
b5+
b6+
b3+
b4+
y5+
y12++
b2+
0
1000
3000
5000
15000
200
400
600
800
1000
1200
